# Enhancing UAV Detection in Surveillance Camera Videos through Spatiotemporal Information and Optical Flow

**DOI:** 10.3390/s23136037

**Published:** 2023-06-29

**Authors:** Yu Sun, Xiyang Zhi, Haowen Han, Shikai Jiang, Tianjun Shi, Jinnan Gong, Wei Zhang

**Affiliations:** Research Center for Space Optical Engineering, Harbin Institute of Technology, Harbin 150001, China; ysun@stu.hit.edu.cn (Y.S.); zhixiyang@hit.edu.cn (X.Z.); jiangshikai@hit.edu.cn (S.J.); shitianjun@stu.hit.edu.cn (T.S.); gongjinnan@hit.edu.cn (J.G.);

**Keywords:** object detection, small target detection, YOLOv5, drone detection, spatiotemporal information

## Abstract

The growing intelligence and prevalence of drones have led to an increase in their disorderly and illicit usage, posing substantial risks to aviation and public safety. This paper focuses on addressing the issue of drone detection through surveillance cameras. Drone targets in images possess distinctive characteristics, including small size, weak energy, low contrast, and limited and varying features, rendering precise detection a challenging task. To overcome these challenges, we propose a novel detection method that extends the input of YOLOv5s to a continuous sequence of images and inter-frame optical flow, emulating the visual mechanisms employed by humans. By incorporating the image sequence as input, our model can leverage both temporal and spatial information, extracting more features of small and weak targets through the integration of spatiotemporal data. This integration augments the accuracy and robustness of drone detection. Furthermore, the inclusion of optical flow enables the model to directly perceive the motion information of drone targets across consecutive frames, enhancing its ability to extract and utilize features from dynamic objects. Comparative experiments demonstrate that our proposed method of extended input significantly enhances the network’s capability to detect small moving targets, showcasing competitive performance in terms of accuracy and speed. Specifically, our method achieves a final average precision of 86.87%, representing a noteworthy 11.49% improvement over the baseline, and the speed remains above 30 frames per second. Additionally, our approach is adaptable to other detection models with different backbones, providing valuable insights for domains such as Urban Air Mobility and autonomous driving.

## 1. Introduction

In recent years, the global drone market has experienced explosive growth, witnessing a significant rise in the overall drone population [1,2]. Drones offer numerous advantages, including low operating costs, exceptional flexibility, and the ability to undertake hazardous missions. As a result, they are exceptionally well-suited for monotonous, harsh, and dangerous work environments, effectively replacing humans in a variety of aerial tasks. Presently, drones have found widespread applications in diverse industries, such as agriculture for crop protection, environmental monitoring, mapping, logistics, and power line inspections [3,4,5]. While these unmanned aerial vehicles offer significant convenience, it is crucial to address the concerns of their potential disorderly and illegal use, which poses significant risks to personal privacy, aviation safety, and public security [6,7]. Particularly considering the changing landscape of international counter-terrorism and security, the effective prevention and control of illicit drone activities have become imperative.

**Figure 1:**
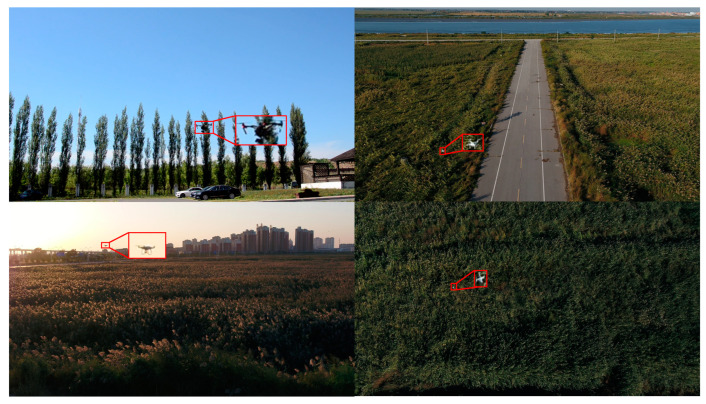
Drone targets in diverse and complex environments.

Currently, the predominant methods for drone monitoring and alerting encompass radar detection [2], acoustic detection [8,9], radio frequency detection [10,11], and electro-optical detection [12,13,14,15]. However, these methods exhibit certain limitations in practical applications [16]. Radar systems, for instance, display a poor detection performance concerning small drones, making it challenging to differentiate between drones and birds. Moreover, their effectiveness diminishes in low-altitude applications, and they suffer from poor electromagnetic compatibility. Acoustic sensors are susceptible to environmental noise and are ill-suited for noisy public environments. Radio frequency scanners also display inadequate detection performance in the face of frequency hopping and signal shielding by drones. In contrast, electro-optical detection methods offer greater adaptability to drones of diverse models and sizes. Furthermore, the rapid advancements in deep learning and computer vision technologies have unlocked significant potential for drone detection methods based on optical/visual systems. These approaches have demonstrated remarkable improvements in detection performance and practicality. However, the surveillance cameras used for drone monitoring are typically deployed in diverse and complex environments, such as urban areas, parks, highways, ports, and airports, resulting in intricate image backgrounds. Additionally, drones often exhibit minimal texture and structural information in the captured images due to their high flying altitudes and considerable distances from the cameras. Consequently, these targets possess small size, low contrast, and weak features, as illustrated in Figure 1. In summary, the limited detection range of surveillance devices, coupled with the complexity and variability of working environments, presents challenges that render drones susceptible to being obscured by the background, thereby impeding the effectiveness of object detection algorithms in identifying drone targets. Therefore, it is imperative to enhance deep learning-based object detection algorithms to improve their performance in detecting such small and weak aerial targets, such as drones.

Research has demonstrated the remarkable sensitivity of the human visual system in perceiving object motion. When humans identify targets, they rely not only on static characteristics but also on temporal variations exhibited by objects [17,18,19]. Motivated by this insight, our primary objective is to integrate temporal information into object detection models to enhance accuracy. To address the high real-time requirements of drone detection tasks, we adopt the YOLOv5s model as our base network, given its lower complexity as a single-stage object detection network. To facilitate the incorporation of continuous sequences of multiple frames as input to the model, we extend its input layer. Additionally, to better exploit the temporal information associated with moving targets, we introduce inter-frame optical flow as an additional input into the model. By leveraging optical flow, which effectively extracts target motion information, we enable a more precise detection of flying drones by seamlessly integrating spatiotemporal information into the model. The experimental results demonstrate that our method achieves excellent detection performance while ensuring high processing speed.

The main contributions of this work can be summarized as follows:

1. Extension of the object detection model’s input to consecutive frames, enabling the model to leverage temporal and spatial information for improved detection performance on dynamic targets;
2. Incorporation of optical flow tensors as input to the model, allowing it to directly acquire motion information between two consecutive frames to more accurately capture features related to drone motion;
3. Verification of the proposed method through comparative experiments to illustrate its effectiveness and superiority.

The paper follows the subsequent structure: Section 2 presents an overview of the current research on drone detection based on deep learning. Section 3 elaborates on the rationale and technical details of our proposed method. In Section 4, we conduct comparative experiments to validate the accuracy of our method. Finally, in Section 5, we present our conclusions.

## 2. Related Work

### 2.1. Single-Frame Image Drone Detection

Object detection methods in the field of deep learning can be classified into two categories: single-stage and two-stage approaches. Single-stage methods, also known as regression-based methods, directly predict bounding boxes and class labels using the model. While single-stage networks are faster, they generally demonstrate a relatively lower accuracy compared to their two-stage counterparts. On the other hand, two-stage methods, referred to as region proposal-based methods, generate initial proposed regions and then conduct classification and regression to derive the final detection results. Despite offering a higher accuracy, two-stage networks often come with the trade-off of slower speed when compared to single-stage networks.

#### 2.1.1. Single-Stage Drone Detection Methods

Object detection algorithms based on single-stage networks, such as SSD [20], YOLO [21], and RetinaNet [22], directly determine the locations and classes of objects from individual frame images. YOLO, as the pioneering single-stage network, partitions the input image into non-overlapping grids, predicting the class and location of an object if its center lies within a grid. SSD introduces multi-scale modules within the network to enhance detection of objects across various sizes. To tackle class imbalance, RetinaNet introduces the Focal Loss. Hassan et al. [23] demonstrated the successful application of YOLOv2 [24] and YOLOv3 [25] in detecting drones using their self-constructed dataset, yielding promising results. However, their dataset primarily comprised large-sized drones, which restricts the effectiveness of their method in detecting distant drone targets and limits its practical utility.

#### 2.1.2. Two-Stage Drone Detection Methods

Object detection algorithms based on two-stage networks follow a process where initially potential candidate boxes that might contain objects are calculated. Subsequently, the network verifies the presence of actual objects within these candidate boxes. Upon detecting an object, the algorithm then proceeds to refine the position of the candidate box and determine its class. Among the notable representatives of this approach is Faster R-CNN [26], which utilizes the Region Proposal Network (RPN) to generate candidate boxes. These boxes are then mapped to fixed-size feature maps using Region of Interest (RoI) Pooling. Finally, the subsequent network performs object classification and position regression. Building upon the foundations of Faster R-CNN, several improved algorithms have been proposed, including Mask R-CNN [27] and Cascade R-CNN [28]. These approaches enhance performance by integrating instance segmentation and cascade detection, respectively. Magoulianitis et al. [29] applied Faster R-CNN to detect drones in upsampled images obtained from a super-resolution network, resulting in an improvement in detection effectiveness to a certain extent. However, this approach encounters a significant decrease in detection speed, which compromises its ability to fulfill high real-time requirements.

### 2.2. Video Drone Detection

The earliest approaches to video object detection drew upon knowledge gained from image object detection. However, the accuracy of object detection in videos is compromised by various factors, including motion blur, occlusion, and out-of-focus instances. To address these challenges, numerous methods have been proposed for aggregating features across video frames. One notable contribution by Zhu et al. [30] is an end-to-end network that leverages temporal coherence at the feature level. This approach enhances per-frame performance, thereby improving video recognition accuracy. Li et al. [31] introduced an interweaved recurrent-convolutional architecture with an efficient bottleneck-LSTM layer to achieve temporal awareness. Another novel method by Wu et al. [32] involves a sequence-level semantics aggregation module that eliminates the need for complex postprocessing techniques. In their work, Wang et al. [33] proposed an aligned spatial–temporal memory network for video object detection. This network utilizes a spatial–temporal memory module to capture long-range dependencies across frames, thereby enhancing detection performance. Deng et al. [34] introduced a single-shot video object detector that integrates feature aggregation into a one-stage detector for object detection in videos. Their approach utilizes the Feature Pyramid Network as the backbone network to produce multiscale features. Chen et al. [35] argued that both local localization information and global semantic information are vital cues for humans to recognize objects in videos. Accordingly, they proposed a memory-enhanced global-local aggregation network.

While these video object detection methods have yielded impressive results, most of them have been evaluated on standard video object detection datasets where the objects occupy a significant portion of the video frame [36]. Consequently, they are not well-suited for detecting drones, which are typically small targets. Addressing the specific characteristics of drones as weak and diminutive objects, [37,38] employed a similar approach by detecting moving drones through background image subtraction and subsequently identifying drones using deep learning classifiers. Another study by Rozantsev et al. [39] utilized two convolutional neural networks to achieve coarse and fine motion stabilization, followed by drone detection using a third network. In contrast, [40] proposes a two-stage segmentation-based approach that incorporates spatiotemporal attention cues instead of relying on region-proposal methods. However, these aforementioned methods are primarily applied to detecting drones within drone videos, while the detection of drones from surveillance camera videos remains largely unexplored.

## 3. Proposed Method

### 3.1. Overall Architecture

**Figure 2:**
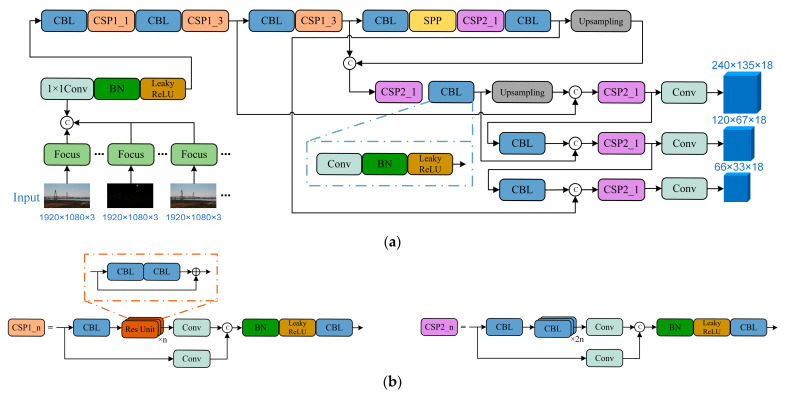

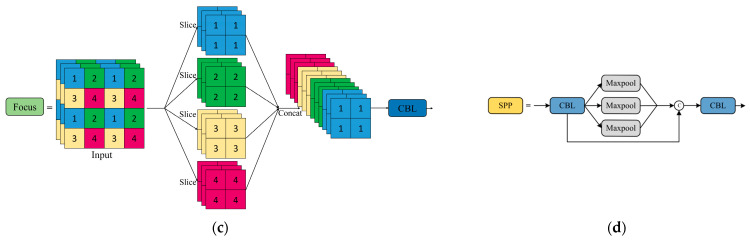
(**a**) Architecture of the proposed method; (**b**) the CSP module; (**c**) the Focus module; (**d**) the SPP module.

The single-stage object detection algorithm offers a lower model complexity, leading to faster processing speeds. Therefore, we selected YOLOv5 as the base network for our drone detection task. YOLOv5 consists of five versions: YOLOv5n, YOLOv5s, YOLOv5m, YOLOv5l, and YOLOv5x, each with an increasing number of parameters. In order to meet the high real-time requirements of drone detection tasks and simultaneously ensure detection performance, we extend the input layer of YOLOv5s. By incorporating a sequence of multiple frames and inter-frame optical flow into the input, we construct an object detection network that effectively utilizes both spatial and temporal information. The overall architecture is presented in Figure 2a,b, illustrating the utilization of CSPDarknet [24] as the feature extraction network, complemented by an FPN [41] structure to enhance the feature information. The CBL structure comprises 3 × 3 convolutional kernels, Batch Normalization, and Leaky ReLU activation functions. To segment the stacked residual blocks, CSPNet [42] adopts the residual structure from ResNet [43]. The Focus module, as shown in Figure 2c, initially employs an interlaced slice operation to partition the input tensor into four equal segments. This process concentrates the spatial information within the channel space and expands the input channel by a factor of four. Subsequently, a batch normalization layer and a non-linear activation function (Leaky ReLU) are applied. The resulting output of the Focus module is a feature map with reduced spatial dimensions but an increased number of channels. By facilitating a balance between computational efficiency and the acquisition of informative features, the Focus module enhances detection accuracy while preserving real-time performance. The SPP module as shown in Figure 2d, employs a parallel structure with pooling kernels of various sizes to capture diverse perceptual fields of the target. Following multiple iterations of CBL and CSPNet processing, three feature maps are generated to sequentially detect objects of small, medium, and large sizes. After extracting informative features from the backbone, the feature layers at various scales are utilized to construct a feature pyramid, bolstering the extraction of target feature information. This process involves upsampling the deep feature map, merging it with the midlevel feature channel, and subsequently performing an upsampling operation to align with the shallow feature channel. By employing a top-down information flow, the preservation of semantic information within the shallow feature maps is achieved.

### 3.2. Extension of the Input Layer for Introducing Temporal Information

The existing methods for general object detection operate by taking a single-frame image as input and producing the locations and classes of objects in that frame. Consequently, these methods can only leverage spatial information contained within each individual frame during processing. However, during drone flights, various features, such as target positions, sizes, and shapes, can change over time. This poses a significant challenge for object detection algorithms relying solely on single-frame images to accurately detect drones, which are characterized by small size, low energy, and limited, variable features. To overcome this challenge, a potential solution is to employ an image sequence consisting of multiple consecutive frames as the input to the detection model, enabling the acquisition and utilization of temporal information. By integrating both spatial and temporal domain information, the detection performance can be enhanced significantly.

**Figure 3:**
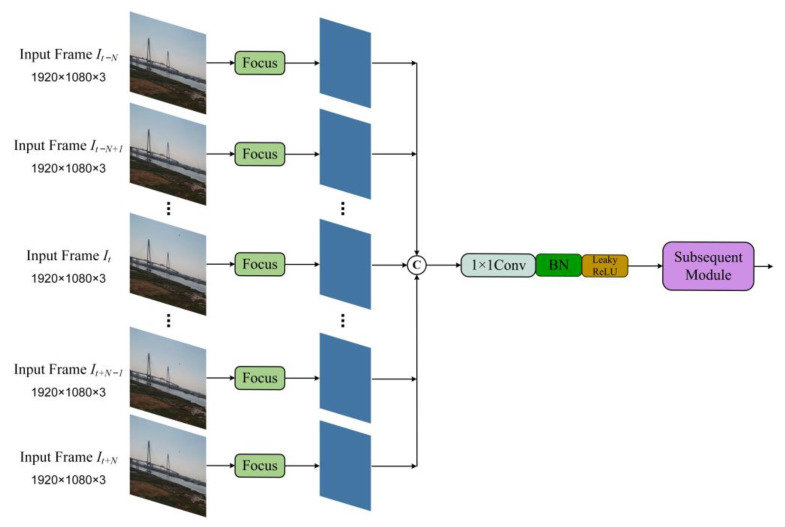
Architecture of the extended input layer for introducing temporal information.

In order to achieve this objective, we propose a restructuring of the input layer of the original YOLOv5s model, expanding it from a single-frame image to an image sequence. Figure 3 illustrates the enhanced structure of the input layer. At time *t*, the input is an image sequence $\left\{I_{j}\left|j=t-N,\cdots ,\right.t,\cdots ,t+N\right\}$ consisting of 2*N* + 1 frames, while the output represents the predicted locations of drone targets in the image *I_t_*. Specifically, after the input sequence of 2*N* + 1 frames undergoes processing with the focus module, the output obtained is connected in series, and the channel dimension is reduced through the 1 × 1 convolution layer, which is followed by a Batch Normalization layer and a Leaky ReLU activation. The subsequent network structure remains consistent with that of the original YOLOv5s model. Notably, the image sequence $\left\{I_{j}\left|j=t-N,\cdots ,\right.t,\cdots ,t+N\right\}$ encompasses both spatial and temporal information. We believe that supplying the network with a continuous series of frames is analogous to enabling the network to emulate the observation process of the flying drones in constant motion by the human visual system. This approach enhances the network’s capability to extract features from small targets and thereby improves detection performance.

### 3.3. Extension of the Input Layer for Introducing Optical Flow

Optical flow leverages pixel matching between consecutive frames to extract vital information regarding pixel motion, including target velocity, direction, and acceleration [44]. Therefore, to augment the detection capability of these small and weak targets by effectively capturing motion-related features associated with drones in the temporal domain, we utilized the Lucas–Kanade (LK) method [45] to estimate the optical flow between two image frames. The optical flow, along with the image sequence, are then input into the object detection network to enhance its performance.

Let P(x,y,t) denote the value of a pixel PI located at image plane coordinates (x,y) at time *t*. According to the brightness constancy assumption of the LK method, which posits that the pixel intensity remains constant as the pixel undergoes motion, we can establish the following equation:

(1) P(x,y,t)=P(x+δx,y+δy,t+δt)

Based on the small motion assumption of the LK method, which considers the object’s movement to be insignificant between two consecutive images, we can expand (1) using Taylor series approximation at the point (x,y,t), while neglecting higher-order terms:

(2) $$\begin{array}{l} P(x+\delta x,y+\delta y,t+\delta t)\approx P(x,y,t)+\frac{\partial P(x,y,t)}{\partial x}\delta x+\frac{\partial P(x,y,t)}{\partial y}\delta y+\frac{\partial P(x,y,t)}{\partial t}\delta t\\ =P(x,y,t)+P_{x}\delta x+P_{y}\delta y+P_{t}\delta t \end{array}$$

By combining (1) and (2) and introducing the variables $u=\frac{\delta x}{\delta t}$ and $v=\frac{\delta y}{\delta t}$, a relationship is revealed:

(3) $$P_{x}u+P_{y}v+P_{t}=0$$

where *u* denotes the velocity of pixel PI along the x-direction and *v* represents its velocity along the y-direction.

The spatial coherence assumption of the LK method states that the motion velocities of all pixels within a small region of size m×m are the same. Based on this assumption, the following system of equations can be derived:

(4) $$\left[\begin{array}{cc} \begin{array}{c} P_{x}{}^{\left(1\right)}\\ P_{x}{}^{\left(2\right)}\\ \ldots \\ P_{x}{}^{\left(N\right)} \end{array} & \begin{array}{c} P_{y}{}^{\left(1\right)}\\ P_{y}{}^{\left(2\right)}\\ \ldots \\ P_{y}{}^{\left(N\right)} \end{array} \end{array}\right]\vec{V}=\left[\begin{array}{c} -P_{t}{}^{\left(1\right)}\\ -P_{t}{}^{\left(2\right)}\\ \ldots \\ -P_{t}{}^{\left(N\right)} \end{array}\right]$$

where $\vec{V}=\left[\begin{array}{c} u\\ v \end{array}\right]$ represents the optical flow vector and superscripts are employed to distinguish between different pixels ($N=m^{2}$).

**Figure 4:**
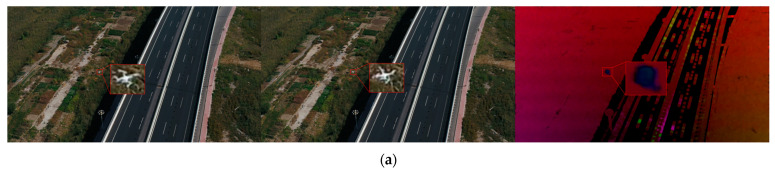

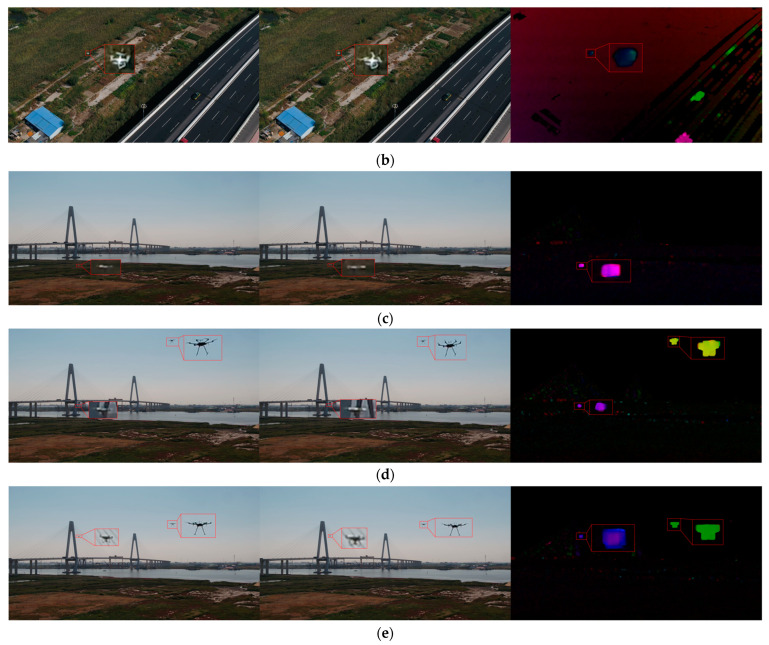
Drone images and inter-frame optical flow. (**a**,**b**) Two frames of images in a highway scene and the optical flow between the two frames; (**c**–**e**) two frames of images in Riverside Park and the optical flow between the two frames.

**Figure 5:**
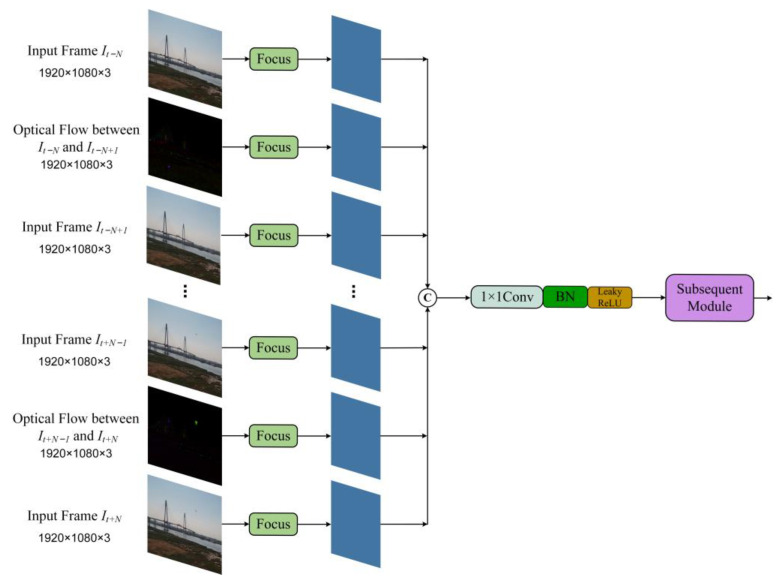
Architecture of the extended input layer for introducing optical flow.

Let $A=\left[\begin{array}{cc} \begin{array}{c} P_{x}{}^{\left(1\right)}\\ P_{x}{}^{\left(2\right)}\\ \ldots \\ P_{x}{}^{\left(N\right)} \end{array} & \begin{array}{c} P_{y}{}^{\left(1\right)}\\ P_{y}{}^{\left(2\right)}\\ \ldots \\ P_{y}{}^{\left(N\right)} \end{array} \end{array}\right]$ and $b=\left[\begin{array}{c} -P_{t}{}^{\left(1\right)}\\ -P_{t}{}^{\left(2\right)}\\ \ldots \\ -P_{t}{}^{\left(N\right)} \end{array}\right]$. By utilizing the least squares method, we obtained the optical flow vector $\vec{V}={\left(A^{T}A\right)}^{-1}A^{T}(-b)$. The phase angle of $\vec{V}$ denotes the motion direction for each pixel within an m×m small local region, while its magnitude indicates the speed of pixel motion. By assigning the phase angle of the optical flow vector to the H channel and the magnitude to the V channel, with all values of the S channel set to 255, we derived a tensor in the HSV color space. This tensor shares the same dimensions as the input image and represents the optical flow between the two images. Let *m* be 3, Figure 4 presents some image instances and optical flow between two images. Observing Figure 4c,d, we can note that the drones located in the lower left region of each image appear to be almost obscured in the background, blending with the surrounding environment. While exhibiting weak features in individual frames, the drone targets are in a state of motion. The visualization of optical flow reveals that the flow computation effectively extracts motion information about the targets. Consequently, by incorporating the optical flow tensors into the model, the deep neural network can directly obtain the motion information of drones between two consecutive frames, thereby enhancing feature extraction for dynamic targets and improving the accuracy and robustness of moving drone detection. To achieve this, we further expanded the input layer of our model, as illustrated in Figure 5. For time *t*, the input consists of an image sequence, $\left\{I_{j}\left|j=t-N,\cdots ,\right.t,\cdots ,t+N\right\}$, with a length of 2*N* + 1, and 2*N* optical flow tensors.

### 3.4. Loss Function

The loss function comprises three essential components: classification loss, bounding box regression loss, and confidence loss. Specifically, the Varifocal loss function [46] was employed to compute the loss for category probability and target confidence score. The CIoU loss function [47] was utilized as the bounding box regression loss. The overall loss function can be expressed as follows:

(5) $$Loss=L_{\mathrm{cls}}+L_{\mathrm{loc}}+L_{\mathrm{obj}}$$

where $L_{\mathrm{cls}}$ denotes the classification loss, $L_{\mathrm{loc}}$ denotes the localization loss, and $L_{\mathrm{obj}}$ denotes the object confidence loss.

## 4. Experiments

### 4.1. Dataset

**Figure 6:**
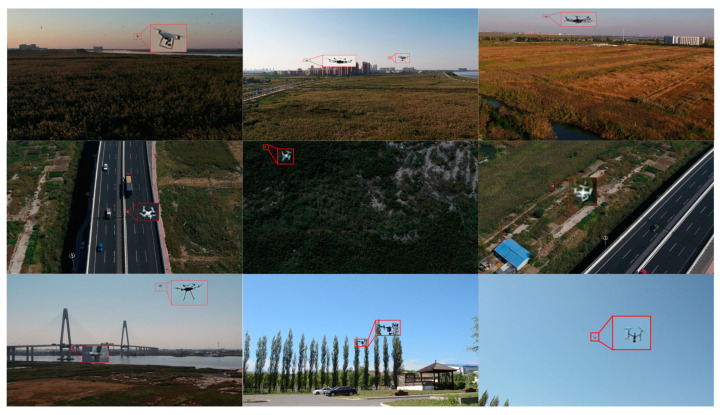
The dataset used in the experiments comprised the following scenes: parks, highways, forests, urban areas, and the sky.

**Figure 7:**
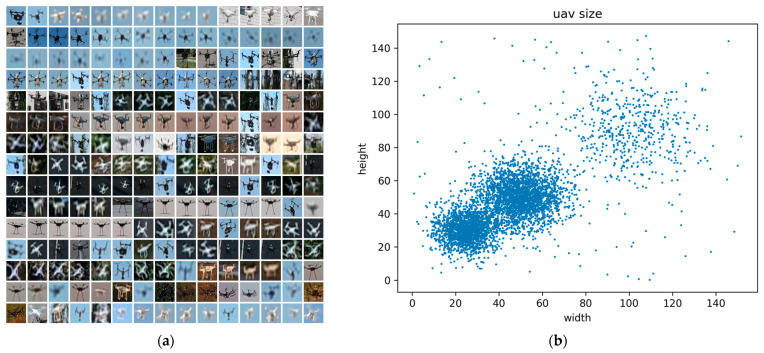
Illustration of the drone targets. (**a**) Displays of small and weak drones; (**b**) size distributions.

The dataset used in this paper for drone detection was constructed based on the drone images captured through a camera. This dataset comprises 4625 images, each with a size of 1920 × 1080 pixels. To create the training, validation, and test sets, we randomly divided the aforementioned images in a ratio of 7:2:1, resulting in 3238 images for the training set, 925 images for the validation set, and 462 images for the test set. Figure 6 showcases part of the dataset, demonstrating diverse and intricate scenes, including parks, highways, forests, urban areas, and the sky, featuring significant cluttered interference. Additionally, Figure 7 presents the target samples and their size distributions.

### 4.2. Implementation Details

All the experiments were conducted on a workstation equipped with an Intel Xeon^®^ Silver 4210R CPU and a NVIDIA RTX3080Ti GPU with a memory size of 12 GB. We conducted our experiments with the Pytorch framework. The input image size in the training process was 1920 × 1080 pixels with a batch size of 8 for 500 epochs. We employed the Adam optimizer with a learning rate of 0.001. The length of the input image sequence was set to 9, i.e., *N* = 5. Since the input was no longer a single-frame image, the mosaic data augmentation technique was not applied.

### 4.3. Evaluation Criteria

The detection performance was evaluated in terms of accuracy and speed. Precision (P), Recall (R), F1-Score (F1), and average precision (AP) were chosen to evaluate the detection accuracy. The inference speed was evaluated by frames per second (FPS), which can evaluate the assess the real-time performance.

Precision is defined as the percentage of correct predictions among all predictions, which can assess the degree of false alarms, while recall measures the degree of missed alarms. It is the percentage of correct predictions among the labeled targets. They are calculated as:

(6) $$P=\frac{TP}{TP+FP}$$

(7) $$R=\frac{\mathrm{TP}}{\mathrm{TP}+\mathrm{FN}}$$

where TP, FP, and FN represent the true positive, false positive, and false negative, respectively.

The F1 score is defined as the harmonic mean of the precision and recall. The AP is defined as the area enclosed by the curve of precision and recall. They are calculated as:

(8) $$F1=\frac{2}{\frac{1}{P}+\frac{1}{R}}$$

(9) $$\mathrm{AP}=\int_{0}^{1}P(R)d(R)$$

### 4.4. Experimental Results and Discussion

To validate the effectiveness and robustness of our proposed method, we conducted a comparative analysis with several representative detectors, including both one-stage and two-stage detectors. Our evaluation focused on detecting small and weak drones in various application scenarios. As presented in Table 1, our method demonstrated competitive performance in terms of both inference speed and detection accuracy. Notably, the two-stage detectors employing coarse-to-fine pipelines do not exhibit superiority in small-drone detection, whereas the one-stage detectors offer an overall speed advantage. Among the various representative methods that do not incorporate temporal information, YOLOv4 achieves the highest detection accuracy compared to other typical networks. In contrast, our method surpassed YOLOv4 with a 3.62% increase in AP and an inference speed that is 1.59 times faster. On the other hand, while the introduction of image sequences and inter-frame optical flow significantly enhances the detection accuracy of YOLOv3 and YOLOv4, the inherent low inference speed of these models becomes further exacerbated. Consequently, expanding the inputs results in an extremely low FPS, rendering them impractical for real-time applications.

**Figure 8:**
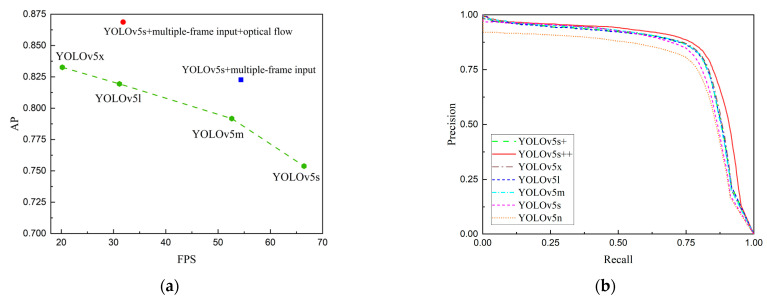
Comparisons with YOLOv5 series. (**a**) AP-FPS curve; (**b**) P-R curve.

Furthermore, we compared our proposed method with other models in the YOLOv5 series to emphasize its advantages in terms of speed and accuracy. Specifically, our method achieved a final AP of 86.87%, representing a noteworthy 11.49% improvement over the baseline. It is important to note that expanding the input to multi-frame image sequences increases the model parameters and reduces the inference speed by 12.08 FPS. Similarly, introducing image sequences and inter-frame optical flow leads to a reduction in inference speed by 34.61 FPS. However, even with these adjustments, the speed remains above 30 FPS. In Figure 8, we provide visualizations of the AP-FPS curve and P-R curve. Our proposed method strikes a balance between accuracy and speed, delivering a detection speed comparable to YOLOv5l while surpassing other YOLOv5 models in terms of accuracy, including the most complex YOLOv5x.

**Figure 9:**
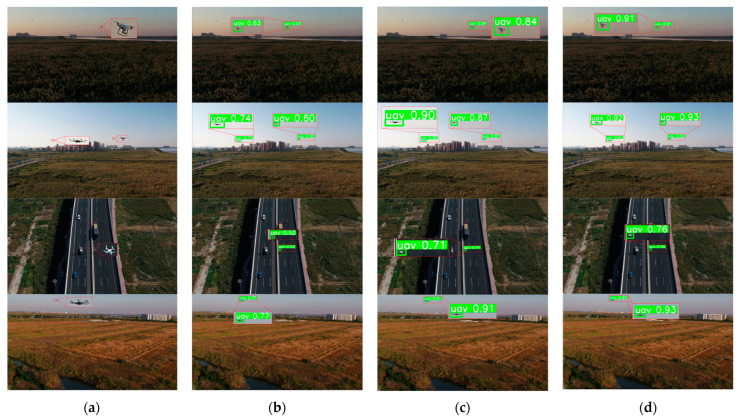
Visualization of the comparative experiments for larger drones. (**a**) Input image; (**b**) YOLOv5s; (**c**) YOLOv5s + multiple-frame input; (**d**) YOLOv5s + multiple-frame input + optical flow.

**Figure 10:**
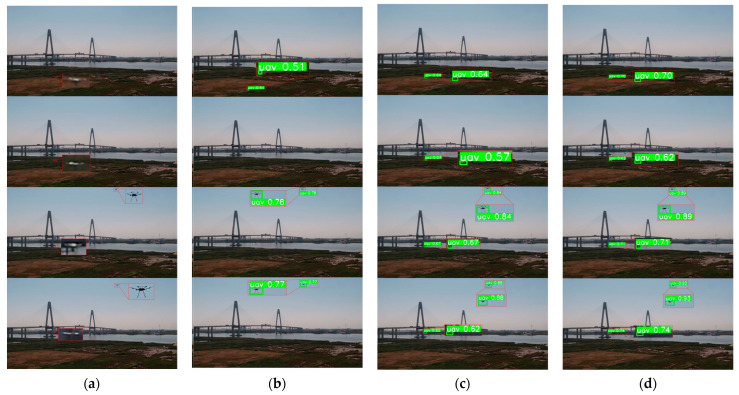
Visualization of the comparative experiments for small and weak drones. (**a**) Input image; (**b**) YOLOv5s; (**c**) YOLOv5s + multiple-frame input; (**d**) YOLOv5s + multiple-frame input + optical flow.

Figure 9 reveals that the baseline model effectively detects targets for larger drones with distinctive features present in the image. Moreover, the proposed model, which integrates multiple-frame inputs and optical flow, further enhanced the confidence scores. In Figure 10, we present the detection results obtained from a sequence of images featuring drone targets with exceedingly weak features, nearly blending into the background. Contrasting the drone located in the upper right portion of Figure 10, the drone positioned in the lower left portion is barely discernible, almost appearing as a horizontal line in individual frames. The performance of the original YOLOv5s is unsatisfactory, as it can scarcely detect such faint targets based solely on single-frame images. The drone situated in the lower left section of the image is only detected in one frame, with a confidence score of 0.51, whereas the drone in the upper right section exhibits superior detection results due to its larger size and richer features. By leveraging the multiple-frame input, the model significantly enhances detection performance for the weak and small drone in motion within the lower left region, surpassing the baseline model. This target becomes detectable in all frames. Furthermore, by incorporating inter-frame optical flow as the input, the proposed method effectively augmented the perception of drones that are nearly indistinguishable from the environment. This approach utilized temporal information and target motion cues, resulting in higher confidence scores. For a more comprehensive presentation of our results, additional detection images are provided in Appendix A.

## 5. Conclusions

In this paper, we proposed a novel method for detecting moving drones in surveillance videos. By simulating the human visual perception of motion, we extended the input of the general object detection network to image sequences and inter-frame optical flow. Drones possess unique characteristics as small, low-energy targets with limited and variable features. By incorporating multiple consecutive frames, the target detection model incorporateed both temporal and spatial information, thereby significantly improving the detection performance of dynamic targets. The introduction of optical flow plays a crucial role in providing pixel motion information, thus augmenting the detection capabilities for small drone targets that may possess limited features within a single frame but are continuously in motion. The experimental results validate the effectiveness of our proposed method, as it successfully strikes a balance between detection performance and inference speed, achieving highly satisfactory outcomes. Specifically, by extending the input to multiple frames, our model achieved a remarkable 6.89% improvement in AP compared to the original YOLOv5s. On this basis, by incorporating optical flow as the input, we observed a notable 11.49% increase in AP, albeit with a 52% decrease in FPS. However, it is important to note that the FPS remains above 30, which meets the high real-time monitoring and alerting requirements. One limitation of our method pertains to its applicability, which is confined to detecting drones in video sequences. The effectiveness of our proposed enhancements relies on the availability of an adequate number of input frames to ensure precise detection. In cases where only a single frame is accessible for input, our network reverts to the original version of YOLOv5 without incorporating temporal context. In future work, we plan to explore additional base network architectures to further enhance the applicability of the method we proposed for expanding the network’s input.

## Figures and Tables

**Table 1:** Comparative experimental results.

Methods	P	R	F1	AP	FPS
Faster-R-CNN	70.13%	63.20%	0.6648	68.75%	27.26
Cascade R-CNN	78.32%	68.08%	0.7284	74.68%	8.63
YOLOv3	78.51%	67.98%	0.7286	73.85%	17.21
YOLOv3+ multiple-frame input	87.59%	75.72%	0.8122	79.43%	6.30
YOLOv3+ multiple-frame input+ optical flow	89.21%	78.36%	0.8343	85.18%	0.87
YOLOv4	83.47%	79.15%	0.8125	83.25%	20.05
YOLOv4+ multiple-frame input	89.28%	84.88%	0.8702	86.71%	10.17
YOLOv4+ multiple-frame input+ optical flow	89.68%	86.24%	0.8792	91.75%	1.48
YOLOv5x	85.92%	80.18%	0.8294	83.26%	20.21
YOLOv5l	83.41%	78.39%	0.8082	81.94%	31.14
YOLOv5m	81.56%	73.47%	0.7730	79.17%	52.63
YOLOv5s	78.77%	66.71%	0.7224	75.38%	66.45
YOLOv5s+ multiple-frame input	86.18%	76.43%	0.8101	82.27%	54.37
YOLOv5s+ multiple-frame input+ optical flow	86.96%	80.67%	0.8369	86.87%	31.84
YOLOv5n	76.38%	63.11%	0.6911	71.51%	75.34

**Figure A1:**
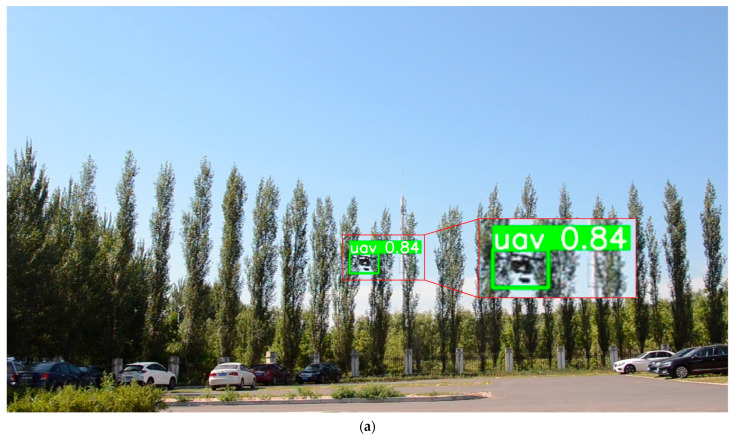

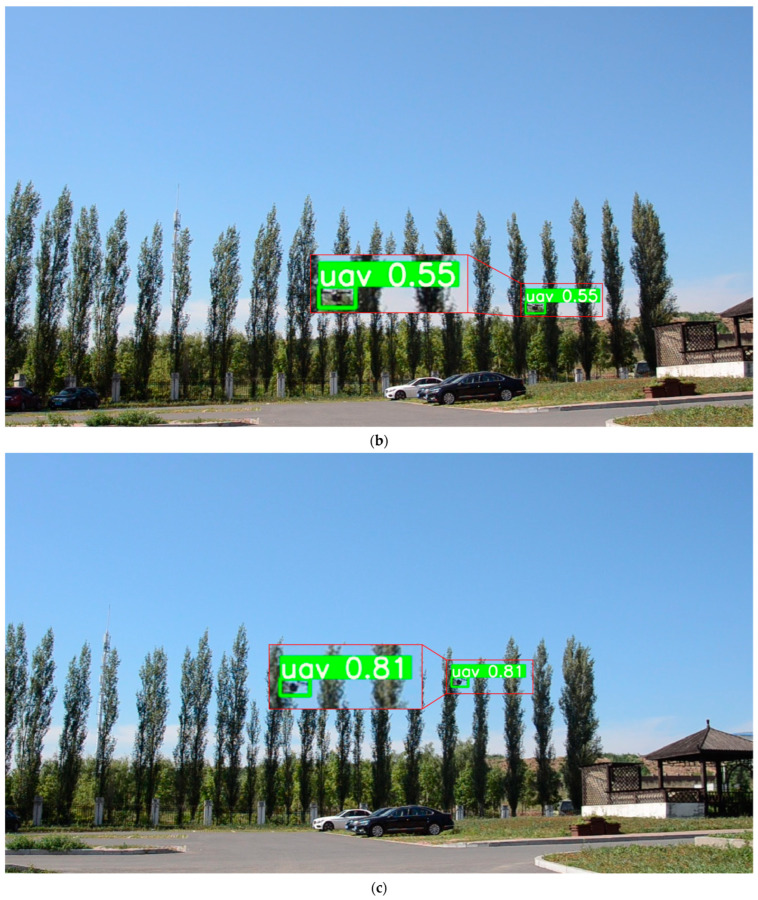

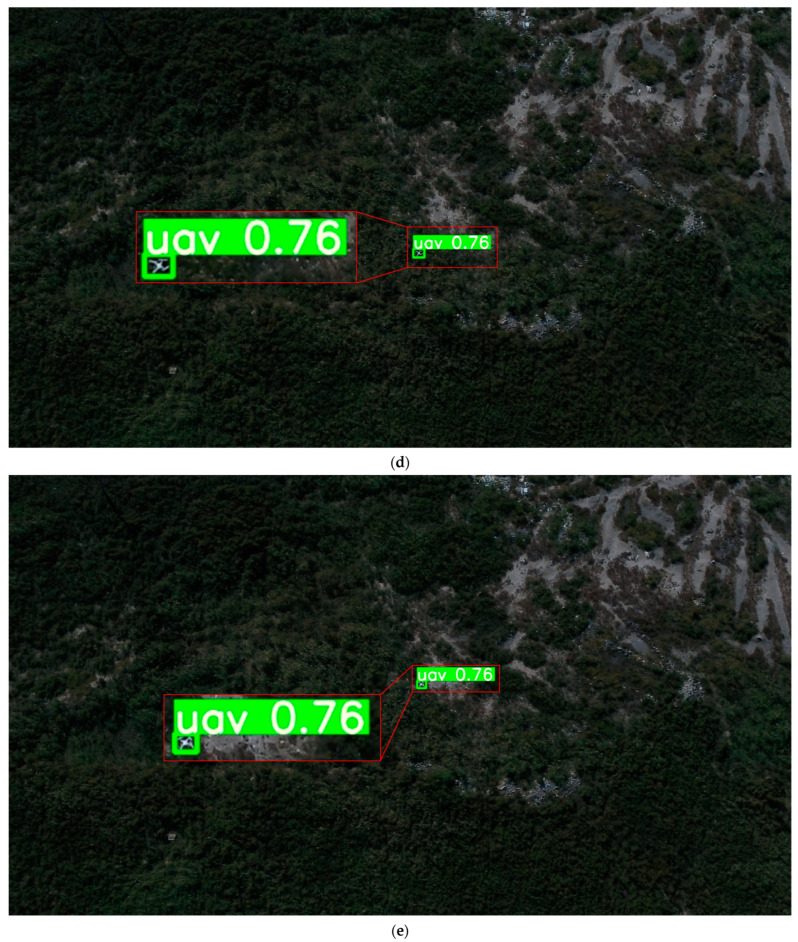

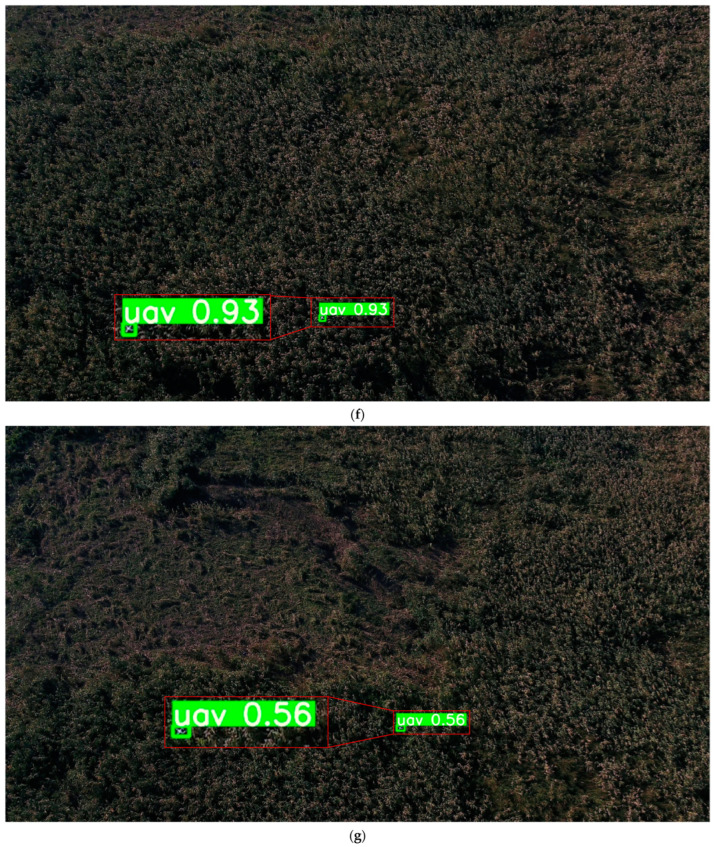

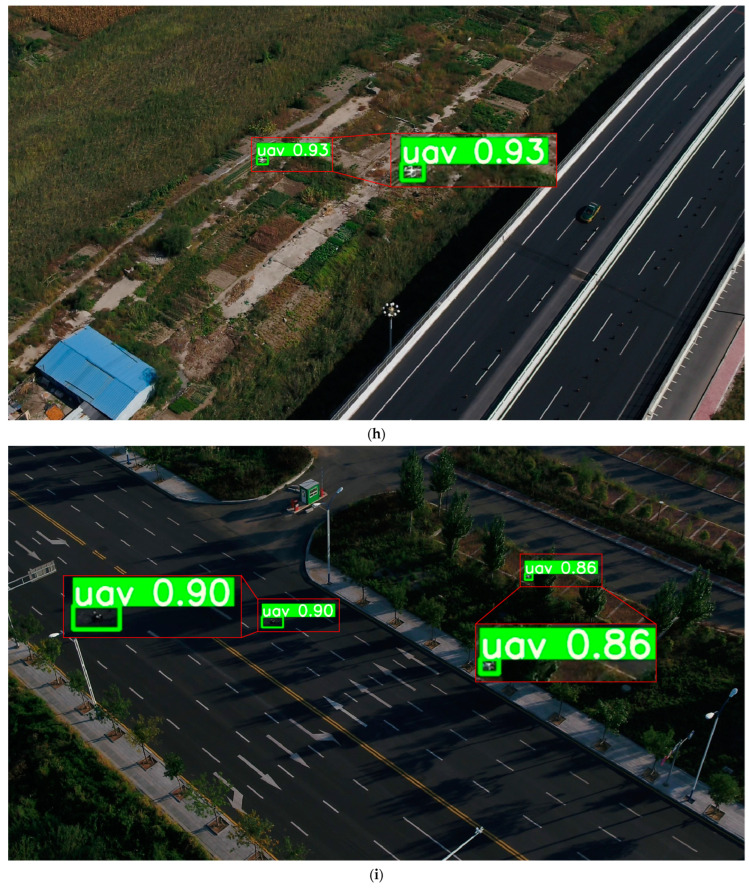

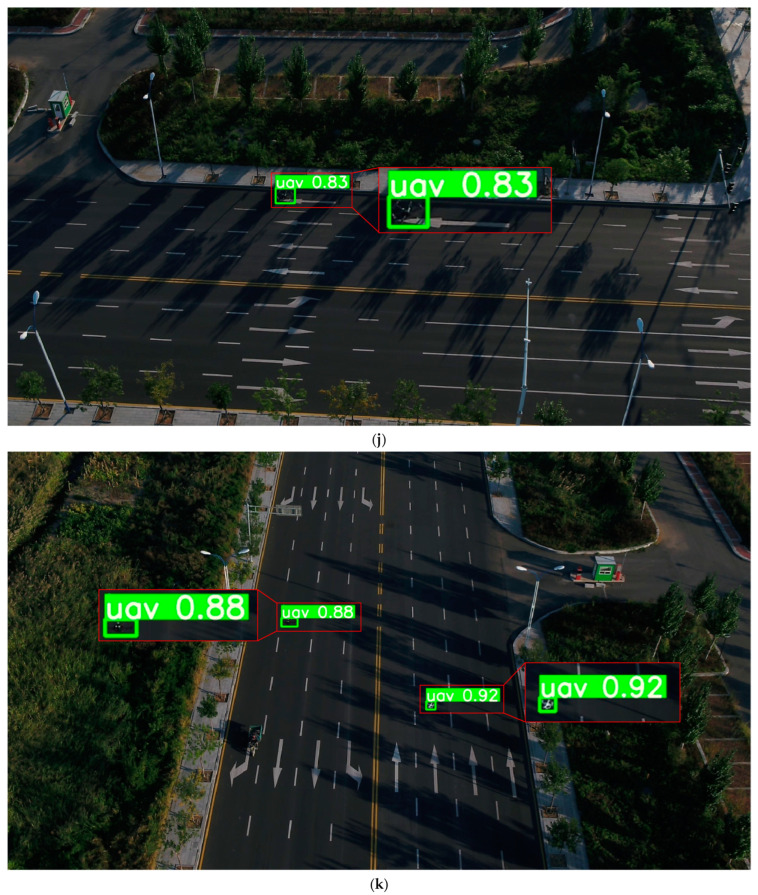
Detection results of small and weak drone targets in surveillance camera videos with bounding boxes and confidence scores. (**a**–**c**) Drone flying over a parking lot; (**d**–**g**) drones flying over a forest; (**h**–**k**) drones flying over a highway.

## Data Availability

The datasets used or analyzed during the current study are available from the corresponding author upon reasonable request.

## Appendix A

Our model effectively detected small and weak drone targets in diverse images, even amidst complex backgrounds and strong cluttered interference. In this section, we present additional detection results to further illustrate our method.
